# High levels of exfoliated fragments following glycocalyx destruction in hemorrhagic fever with the renal syndrome are associated with mortality risk

**DOI:** 10.3389/fmed.2023.1096353

**Published:** 2023-04-17

**Authors:** Hong Du, Haifeng Hu, Jing Li, Xiaoyan Wang, Hong Jiang, Jianqi Lian, Ying Zhang, Pingzhong Wang

**Affiliations:** Department of Infectious Diseases, Second Affiliated Hospital of Air Force Medical University, Xi’an, China

**Keywords:** hemorrhagic fever with renal syndrome, glycocalyx, heparan sulfate, hyaluronic acid, chondroitin sulfate

## Abstract

**Background:**

The glycocalyx is a gel-like structure that covers the luminal side of vascular endothelial cells. It plays an important role in maintaining the integrity of the vascular endothelial barrier structure. However, the presence or absence of glycocalyx destruction in hemorrhagic fever with renal syndrome (HFRS) and its specific mechanism and role is still unclear.

**Methods:**

In this study, we detected the levels of exfoliated glycocalyx fragments, namely, heparan sulfate (HS), hyaluronic acid (HA), and chondroitin sulfate (CS), in HFRS patients and investigated their clinical application value on the evaluation of disease severity and prognosis prediction.

**Results:**

The expression of exfoliated glycocalyx fragments in plasma was significantly increased during the acute stage of HFRS. The levels of HS, HA, and CS in HFRS patients during the acute stage were significantly higher than in healthy controls and convalescent stages of the same type. HS and CS during the acute stage gradually increased with the aggravation of HFRS, and both fragments showed a significant association with disease severity. In addition, exfoliated glycocalyx fragments (especially HS and CS) showed a significant correlation with conventional laboratory parameters and hospitalization days. High levels of HS and CS during the acute phase were significantly associated with patient mortality and demonstrated an obvious predictive value for the mortality risk of HFRS.

**Conclusion:**

Glycocalyx destruction and shedding may be closely associated with endothelial hyperpermeability and microvascular leakage in HFRS. The dynamic detection of the exfoliated glycocalyx fragments may be beneficial for the evaluation of disease severity and prognosis prediction in HFRS.

## Introduction

Hemorrhagic fever with renal syndrome (HFRS) is an acute, natural-focus disease caused by the Hantavirus. It is characterized by fever, shock, hemorrhage, and acute kidney injury (AKI) (1). The Hantaan virus (HTNV) is the most prominent serotype of Hantavirus in China (especially in the Shaanxi province) and is usually accompanied by a more severe clinical course and a higher fatality rate (2). HFRS has the characteristics of a systemic inflammatory response syndrome, and the pathological basis for the clinical manifestations of HFRS is increased vascular endothelial permeability resulting from various mechanisms (3, 4). HFRS patients usually suffer from five typical clinical courses successively, including the febrile, hypotensive, oliguric, diuretic, and convalescent phases. In some critically ill patients, the febrile, hypotensive shock, and oliguric phases can overlap, which might result in refractory shock, acute respiratory distress syndrome, encephalopathy, and severe coagulation dysfunction (5, 6). To date, there has been no specific antiviral therapy for HFRS, and supportive management is still the dominant treatment principle (7). Due to the insufficient predictive efficiency of conventional laboratory parameters for disease severity and prognosis because of the complicated pathophysiological mechanism, it is vital to explore novel biomarkers to ensure early, systematic, and timely interventions (8).

As a hydrated gel-like multilayered mesh coating the luminal surface of vascular endothelial cells, the glycocalyx is an important structure composed of sulfated proteoglycans and glycosaminoglycans, and the core proteins covalently bind glycosaminoglycans to maintain the integrity of the endothelial barrier structure and regulate vascular permeability (9, 10). Heparan sulfate (HS), hyaluronic acid (HA), and chondroitin sulfate (CS) are major components of glycosaminoglycans, and HS and CS are situated in the core protein of proteoglycans, including syndecans and glypicans (9, 11). As a neutral component of glycosaminoglycans, the HA can bind to the cell surface protein CD44 to maintain the stability of the glycocalyx structure (12). The complex and charge structure of the glycocalyx, which forms a mechanical and electrostatic barrier, is capable of sieving and repelling the negatively charged macromolecules such as plasma proteins and blood cells such as leukocytes, erythrocytes, and platelets (10, 13).

Because the glycocalyx is a highly fragile and unstable layer, it is extremely susceptible to degradation by different stimuli, including fluctuating shear stress from the blood flow and inflammatory mediators released by inflammation, trauma, infection, hypoxia, and other pathological processes (9, 11–15). The shedding and degradation of the glycocalyx structure trigger the destruction of the endothelial barrier and increase vascular permeability (9, 10). Furthermore, the exfoliated fragments of the glycocalyx also provoke the rolling, adhesion, migration, and activation of leukocytes, leading to the release of downstream inflammatory cytokines and damage-associated molecular patterns (DAMPs) (16, 17). Activated pro-inflammatory cascades, in turn, aggravate the breakdown of the endothelial barrier and increase the vascular permeability, further promoting the pathophysiological progression of microvascular leakage (9, 10, 15, 16).

Previous studies have shown that glycocalyx destruction induced by pathogenic virus infection is associated with the pathophysiological progress of “microvascular leakage” in dengue, AIDS, and COVID-19 (18–23), while few studies have reported the destruction of glycocalyx caused by the Hantavirus. Our previous studies showed that the plasma levels of soluble CD138 (also called syndecan-1, a component of syndecans in the glycocalyx) were significantly increased during the acute stage in HFRS patients infected with HTNV, which indicated that there may be glycocalyx destruction in vascular endothelial cells in patients with HFRS and that the levels of soluble CD138 may reflect the severity of the disease to a certain extent (24). Connolly-Andersen et al. found that syndecan-1 was significantly associated with the levels of thrombocytes and albumin, decreased blood pressure, disease severity, and patient’s prognosis in nephropathia epidemica caused by the Puumala hantavirus (25). However, the dynamic changes of the exfoliated glycocalyx components (HS, HA, and CS) during the clinical course and their roles in vascular endothelial injury in HFRS are still unclear. In this study, we prospectively detected the plasma levels of HS, CS, and HA in patients with HFRS and investigated their predictive values for disease severity and prognosis.

## Materials and methods

### Participants

Patients with HFRS who were treated at the Second Affiliated Hospital of the Air Force Medical University from October 2011 to December 2013 were prospectively recruited. All enrolled patients underwent serological diagnosis and showed positivity for HFRS-specific IgM and IgG and were consistent with the typical clinical manifestations of HFRS. In addition, 23 healthy volunteers were recruited as controls.

### Grouping criteria and definitions

According to the clinical classification criteria of the HFRS (26), the enrolled patients were divided into the following four groups: (1) mild-type group: patients with mild renal impairment without oliguria and hypotension; (2) moderate-type group: patients with obvious symptoms of effusion (bulbar conjunctiva), hypotension, hemorrhage (mucocutaneous petechiae), and AKI with typical oliguria; (3) severe-type group: patients with severe uremia, effusion (bulbar conjunctiva and either peritoneum or pleura), hemorrhage (ecchymosis and hematoma), hypotension, and AKI with oliguria (urine output: 100–400 ml/day) ≤5 days or anuria (urine output: <100 ml/day) ≤2 days; and (4) critical-type group: patients with one or more of the following complications compared with the severe-type patients: refractory shock (≥2 days), visceral hemorrhage, heart failure, pulmonary edema, brain edema, severe secondary infection, and severe AKI with either oliguria (urine output: 100–500 ml/day) >5 days or anuria (urine output: <100 ml/day) >2 days. Furthermore, the clinical course was divided into the acute stage (including the febrile, hypotensive, and oliguric phases) and the convalescent stage (including the diuretic and convalescent phases) based on the clinical characteristics of the disease (3, 5, 6, 8, 24). The patients were first followed up 28 days after discharge. The prognosis in this study was defined as survival or death (during hospitalization or during the duration of follow-up).

### Materials

A total of 154 venous blood samples in EDTA-anticoagulant tubes (including 88 samples during the acute stage and 66 samples during the convalescent stage) were collected from the enrolled patients. The samples were centrifuged (4°C, 1,200 *g*, 10 min) to extract the plasma. A total of 23 samples were obtained from healthy controls.

### Methods

Plasma HS, HA, and CS levels were detected using commercially available ELISA kits (Quantikine, XiTang Inc., Shanghai, China). All samples were tested in duplicate, and the mean concentration was used for further analysis. Clinical laboratory examinations (routine blood tests, biochemical tests, and coagulation tests) were performed in the Central Laboratory Department of the hospital. Demographic information, hospitalization days, clinical prognosis, and conventional laboratory parameters (with the same sampling time as the above-mentioned 154 blood samples) were collected and summarized from the electronic medical records.

### Statistical analysis

The normality of the continuous variables was calculated using the Shapiro–Wilk test. Normally distributed variables were presented in the form of a mean with standard deviations and compared using the Student’s *t*-test or a one-way analysis of variance. Non-normally distributed variables were presented in the form of the median (25th percentile, 75th percentile) and compared using the Mann–Whitney *U*-test or Kruskal–Wallis *H*-test. The levels of plasma exfoliated fragments of glycocalyx (HS, HA, and CS) between the acute stage and the convalescent stage were compared using the Wilcoxon matched-pairs signed-rank test. Categorical variables were presented in the form of numbers (percentages) and compared using the chi-squared test. The relationship between the level of exfoliated fragments and conventional laboratory parameters was analyzed by Spearman’s non-parametric correlation test. The receiver operating characteristic (ROC) curve analysis was used to evaluate the predictive efficacy of HS, CS, HA, and conventional laboratory parameters for the prognosis (death) of HFRS, and the area under the ROC curve (AUC) was further compared using the Mann–Whitney *U*-test. The association between HS, CS, and HA and mortality was assessed using the Kaplan–Meier survival analysis. For a two-tailed test, a *p*-value of <0.05 was considered to indicate statistically significant differences. All the statistical analyses and graphing were performed using SPSS Statistics 23.0 and GraphPad Prism 8.

## Results

### Demographic characteristics of The enrolled patients

A total of 91 HFRS patients [20 (21.98%) women and 71 (78.02%) men] with a mean age of 43.18 ± 13.96 years were enrolled in this study. According to the clinical classification criteria of HFRS (26), 19 cases were classified as mild type, 29 as moderate type, 22 as severe type, and 21 as critical type. Of the enrolled patients, 13 (14.29%) critical-type patients died. There was no significant difference in age distribution, sex, and blood sampling time of the acute stage among the different clinical types and healthy controls, as well as between the surviving and non-surviving patients (*p* > 0.05; Table 1). Of the enrolled patients, the median length of hospital stay was 14 days, which increased gradually with the aggravation of the disease. However, the median length of hospital stay for deceased patients was only 5 days, which was attributed to the fact that death usually occurred soon after admission (Table 1).

**Table 1:** Demographic, clinical, and laboratory parameters of the study population.

	Mild (*n* = 19)	Moderate (*n* = 29)	Severe (*n* = 22)	Critical (*n* = 21)	^a^HC (*n* = 23)	*χ^2^*/*F*/*H*	*P*	Alive (*n* = 78)	Dead (*n* = 13)	*χ^2^*/*t*/*Z*	*P*
Gender											
Men	14 (73.7%)	22 (75.9%)	18 (81.8%)	17 (81.0%)	16 (69.6%)	1.274	0.866	61 (78.2%)	10 (76.9%)	0.011	0.918
Women	5 (26.3%)	7 (24.1%)	4 (18.2%)	4 (19.0%)	7 (30.4%)			17 (21.8%)	3 (23.1%)		
Age, years	39.47 ± 15.72	42.62 ± 13.43	40.64 ± 11.61	48.38 ± 10.55	41.65 ± 12.46	2.516	0.064	42.44 ± 13.27	46.62 ± 15.89	−1.454	0.183
^b^Blood sampling, days	6.3 ± 1.8	6.2 ± 1.5	6.4 ± 1.4	5.4 ± 1.4	—	1.684	0.177	6.2 ± 1.3	5.5 ± 1.6	2.097	0.068
Hospitalization days	10.0 (9.0–11.0)	13.0 (12.0–15.0)	20.0 (16.0–23.0)	23.0 (6.0–27.0)	—	48.469	<0.001	14.0 (11.0–21.0)	5.0 (2.0–10.0)	−4.802	<0.001
CS, ng/mL											
Acute stage	11.47 (9.64–16.32)	27.05 (13.50–62.85)	38.68 (18.15–154.18)	97.57 (48.45–581.50)	14.00 (7.15–21.35)	49.810	<0.001	27.21 (12.59–71.70)	116.17 (36.87–581.50)	−2.498	0.013
Convalescent stage	8.59 (5.19–10.69)	8.11 (5.98–15.34)	9.64 (6.15–13.66)	6.62 (5.91–15.30)		6.543	0.162	—	—	—	—
HS, ng/mL											
Acute stage	20.44 (9.73–54.20)	37.75 (25.55–82.95)	52.12 (20.95–101.77)	110.73 (37.85–135.30)	4.24 (2.58–7.04)	85.208	<0.001	46.41 (20.36–86.86)	111.42 (37.85–135.30)	−2.669	0.008
Convalescent stage	6.91 (4.61–8.19)	6.77 (4.78–10.84)	5.65 (3.60–10.06)	6.58 (4.01–12.07)		11.480	0.022	—	—	—	—
HA, ng/mL											
Acute stage	385.13 (202.71–1386.77)	456.21 (325.81–2162.75)	399.72 (202.13–583.96)	407.32 (168.68–645.43)	50.82 (31.42–65.91)	71.177	<0.001	426.42 (279.66–1200.10)	349.35 (119.59–676.25)	−1.626	0.104
Convalescent stage	40.53 (15.29–201.61)	99.03 (50.02–244.42)	47.03 (20.84–127.62)	120.69 (72.11–196.22)		12.760	0.013	—	—	—	—

a HC, healthy controls.

b Blood sampling: the interval from the onset of illness to the day of blood collection during the acute stage.

### Levels of plasma HS, HA, and CS in patients with HFRS

**Figure 1:**
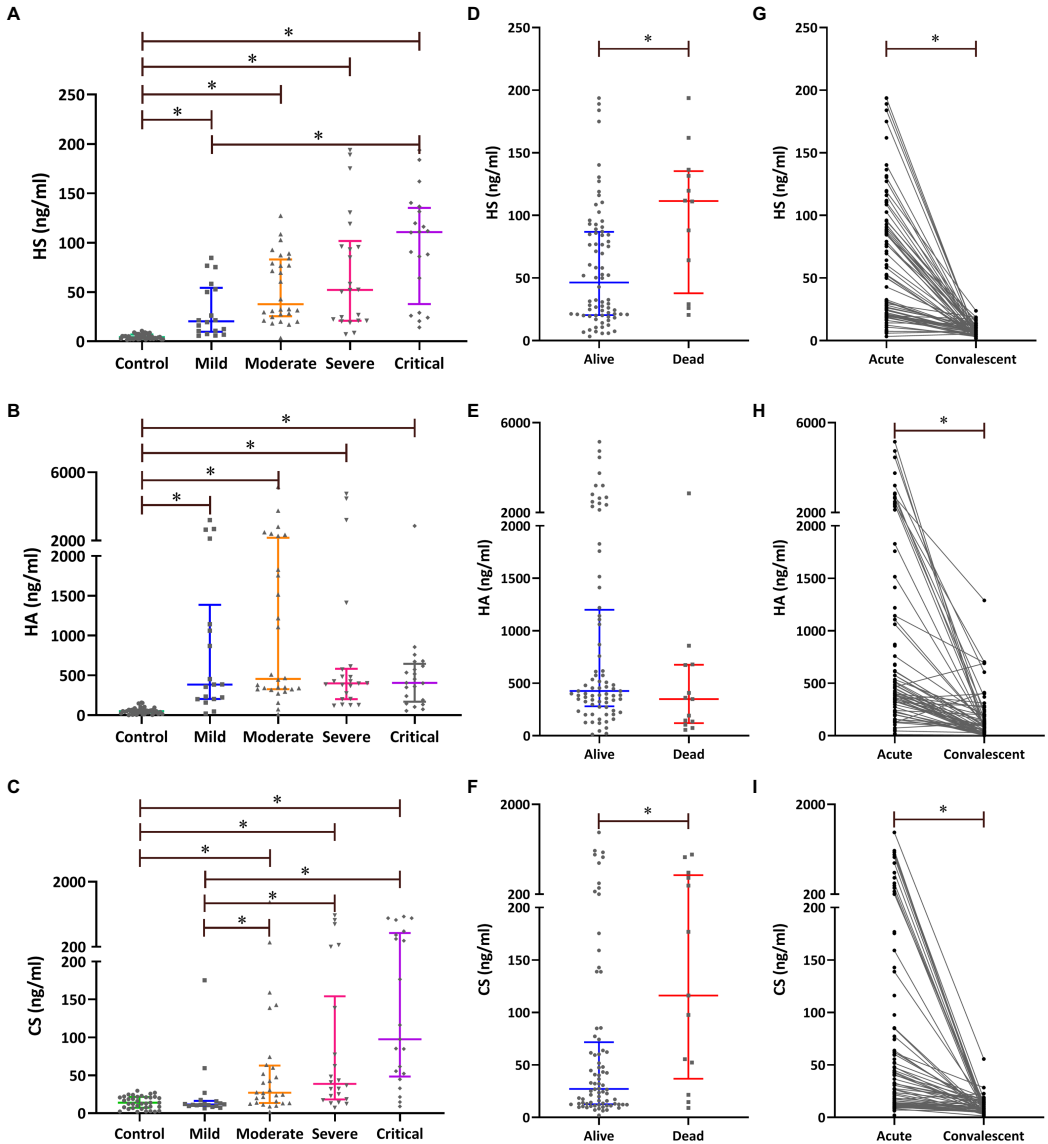
Plasma levels of exfoliated glycocalyx components [heparan sulfate (HS), hyaluronic acid (HA), and chondroitin sulfate (CS)] in patients with HFRS. The levels of plasma HS **(A)**, HA **(B)**, and CS **(C)** during the acute stage were compared using the Kruskal–Wallis *H*-test; pairwise comparisons among the five groups were performed using the Nemenyi rank test. The levels of plasma HS **(D)**, HA **(E)**, and CS **(F)** during the acute stage were compared between the survivors and non-survivors using the Mann–Whitney *U*-test. The differences in plasma HS **(G)**, HA **(H)**, and CS **(I)** between the acute and the convalescent stages were compared using the Wilcoxon matched-pairs signed-ranks test. *p < 0.05.

The levels of plasma HS and CS during the acute stage showed an increasing trend with disease progression, and the highest expression of plasma HS and CS was found in the critical-type patients (*p* < 0.05), both of which presented a significant correlation with disease severity (Table 1). Patients with more severe diseases and non-survivors had higher levels of plasma HS and CS during the acute stage (Figure 1). The levels of plasma HS, HA, and CS in patients with HFRS were all significantly higher during the acute stage than in the healthy controls. Furthermore, the levels of plasma HS, HA, and CS rapidly decreased when the convalescent stage was reached (Figure 1).

### Correlations of HS, HA, and CS with conventional laboratory parameters and hospital stay

**Figure 2:**
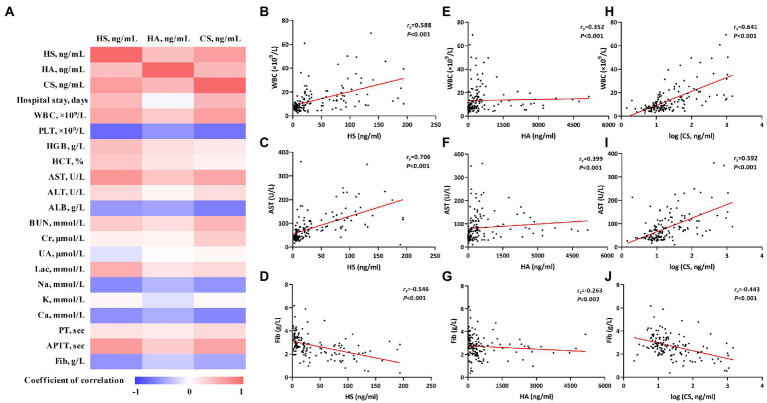
The correlations of heparan sulfate (HS), hyaluronic acid (HA), and chondroitin sulfate (CS) with conventional laboratory parameters. Correlations were calculated using Spearman rank correlation analysis. The correlation coefficients were represented by different shades of color in panel **(A)**; the red and blue colors represent positive and negative correlations, respectively. The correlations of HS, HA, and CS with individual laboratory parameters [white blood cells (WBC), aspartate transaminase (AST), and Fib] are shown in panels **(B)** through **(J)**.

The correlations between HS, HA, and CS and conventional laboratory parameters were further analyzed. Spearman’s correlation analysis indicated that HS, HA, and CS positively correlated with each other and also with conventional laboratory parameters such as white blood cells (WBC), aspartate transaminase (AST), and activated partial thromboplastin time (APTT) and negatively correlated with platelets (PLT), albumin (ALB), and fibrinogen (Fib; Table 2 and Figure 2).

**Table 2:** Correlations between heparan sulfate (HS), hyaluronic acid (HA), and chondroitin sulfate (CS) and conventional laboratory parameters.

Laboratory parameters	HS, ng/mL		HA, ng/mL		CS, ng/mL	
	*r*_s_	*P*-value	*r*_s_	*P*-value	*r*_s_	*P*-value
HS, ng/mL	1.000	−	0.432	<0.001	0.657	<0.001
HA, ng/mL	0.432	<0.001	1.000	−	0.483	<0.001
CS, ng/mL	0.657	<0.001	0.483	<0.001	1.000	−
^a^Days in the hospital	0.467	<0.001	−0.037	0.748	0.485	<0.001
WBC, ×10^9^/L	0.588	<0.001	0.352	<0.001	0.641	<0.001
PLT, ×10^9^/L	−0.765	<0.001	−0.545	<0.001	−0.706	<0.001
HGB, g/L	0.431	<0.001	0.233	0.004	0.154	0.057
HCT, %	0.379	<0.001	0.185	0.022	0.099	0.223
AST, U/L	0.706	<0.001	0.399	<0.001	0.592	<0.001
ALT, U/L	0.265	0.001	0.068	0.413	0.235	0.004
ALB, g/L	−0.524	<0.001	−0.466	<0.001	−0.655	<0.001
BUN, mmol/L	0.353	<0.001	0.227	0.005	0.487	<0.001
Cr, μmol/L	0.112	0.170	0.079	0.332	0.340	<0.001
UA, μmol/L	−0.154	0.058	0.018	0.829	0.065	0.427
Lac, mmol/L	0.549	<0.001	0.184	0.106	0.258	0.023
K^+^, mmol/L	0.058	0.483	−0.163	0.049	0.052	0.529
Na^+^, mmol/L	−0.595	<0.001	−0.371	<0.001	−0.544	<0.001
Ca^2+^, mmol/L	−0.571	<0.001	−0.418	<0.001	−0.610	<0.001
PT, sec	0.190	0.028	0.140	0.103	0.251	0.003
APTT, sec	0.670	<0.001	0.341	<0.001	0.609	<0.001
Fib, g/L	−0.546	<0.001	−0.263	0.002	−0.443	<0.001

a Data on the number of days spent in the hospital were calculated from the survivors.

As death usually occurs soon after admission in some critical-type patients, only the length of hospital stay of the survivors was used for the correlation analysis. HS and CS showed significant correlations with the length of hospital stay (Figure 2). More detailed information about the correlation analysis is shown in Table 2.

### ROC curves for predictive efficacy and hazard ratio of death

**Figure 3:**
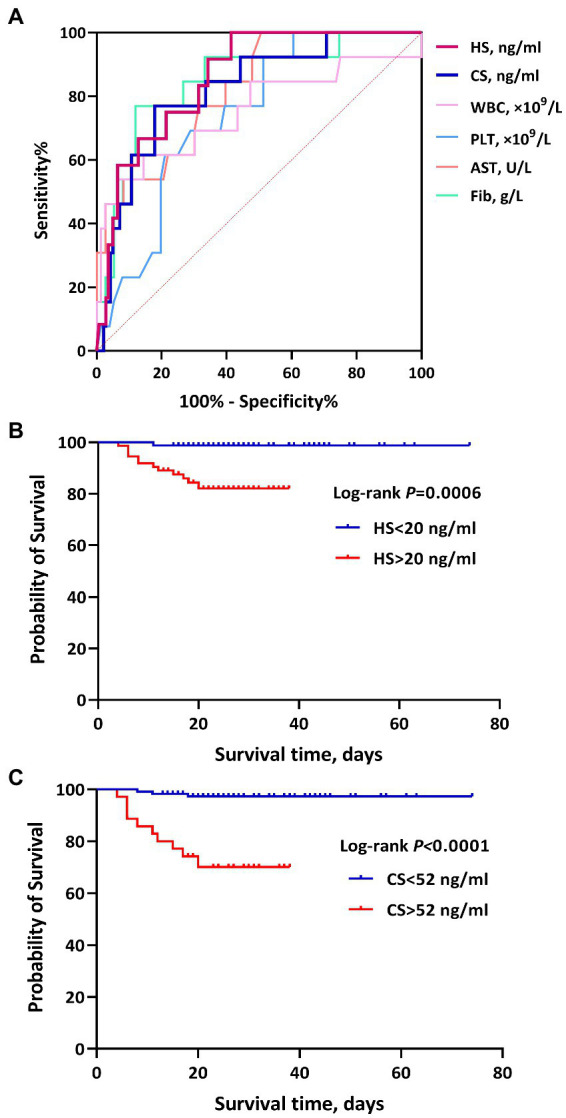
The ROC curves and survival curves of heparan sulfate (HS) and chondroitin sulfate (CS) for the prognosis (death) of hemorrhagic fever with renal syndrome (HFRS). **(A)** shows the predictive efficacy of HS, CS, white blood cells (WBC), platelets (PLT), aspartate transaminase (AST), and Fib for the prognosis (death) in patients with HFRS. HS and CS showed significant predictive values, with an AUC of 0.871 and 0.822, respectively. **(B)** and **(C)** represent the association of HS and CS with mortality in HFRS, respectively. Higher levels of HS and CS showed a higher risk of mortality.

Given the aforementioned results of the intergroup comparisons and correlation analyses, it is tentatively speculated that the levels of HS and CS would be significantly related to the disease severity and prognosis of HFRS. Subsequently, the ROC curves were used to evaluate the predictive values of HS and CS for the prognosis. Both HS and CS exhibited obvious predictive value for the prognosis (death), with an AUC of 0.871 (95%CI: 0.807–0.935, *p* < 0.001) and 0.822 (95%CI: 0.709–0.935, *p* < 0.001), respectively. HS and CS demonstrated comparable predictive efficacy with WBC, PLT, AST, APTT, and other conventional laboratory parameters, with a sensitivity of 100 and 76.92%, respectively, for predicting the prognosis (death; Table 3 and Figure 3).

**Table 3:** Predictive efficacy of heparan sulfate (HS), hyaluronic acid (HA), and chondroitin sulfate (CS) and conventional laboratory parameters.

	AUC (95% CI)	*P*-value	Cut-off value	Sensitivity	Specificity	^a^Comparison of AUC	
						*Z*	*P*-value
HS, ng/mL	0.871 (0.807–0.935)	<0.001	20.42	100.00%	62.44%	−	−
HA, ng/mL	0.549 (0.406–0.692)	0.558	−	−	−	−	−
CS, ng/mL	0.822 (0.709–0.935)	<0.001	51.99	76.92%	82.14%	0.635	0.526
WBC, ×10^9^/L	0.752 (0.576–0.927)	0.004	31.53	53.85%	93.42%	1.621	0.098
PLT, ×10^9^/L	0.742 (0.620–0.863)	0.005	42	48.68%	92.31%	1.705	0.087
ALB, g/L	0.762 (0.583–0.941)	0.003	24.95	86.84%	69.23%	1.534	0.112
AST, U/L	0.821 (0.707–0.935)	<0.001	85	100.00%	49.32%	0.638	0.526
APTT, sec	0.884 (0.766–1.000)	<0.001	51.6	76.92%	92.00%	0.265	0.753
Fib, g/L	0.849 (0.733–0.966)	<0.001	1.642	88.00%	76.92%	0.535	0.602
Cr, μmol/L	0.557 (0.397–0.716)	0.515	−	−	−	−	−
BUN, mmol/L	0.569 (0.388–0.750)	0.430	−	−	−	−	−

a Comparison of AUC: The AUC of each parameter was compared with HS using the Mann–Whitney *U*-test.

Using death/survival as the terminal event, lengths of illness (duration from illness onset to discharge/death) as the survival time, and grouping based upon the cutoff values of ROC curve analyses, the results of the Kaplan–Meier survival analysis with the log-rank test showed that high levels of HS (>20 ng/ml) and CS (>52 ng/ml) during the acute stage were significantly associated with death in HFRS patients, with a hazard ratio (HR) of 14.64 (4.91–43.65) and 12.64 (3.32–48.18), respectively (Figure 3).

## Discussion

As a layer of negatively charged gel-like structure overlying the surface of endothelial cells, the glycocalyx consists of proteoglycans with one or more side chains of glycosaminoglycans, and its degradation increases transcellular transport without affecting endothelial cell junctions (27). Destruction and shedding of the glycocalyx have been found to be associated with an increase in vascular permeability in many diseases with “vascular leakage” features, such as sepsis, trauma, and bacterial infection. Because glycocalyx degradation occurs in certain pathological conditions, circulating levels of glycocalyx degradation products have been recognized as having diagnostic or prognostic value. In addition to their roles as biomarkers, certain glycocalyx fragments may also serve as pathogenic factors capable of inducing endothelial hyperpermeability and microvascular leakage during inflammation (28). Increased vascular permeability is the most prominent pathophysiological feature of HFRS, the mechanism of which is not yet fully understood. The predominant causes of death in HTNV-induced HFRS patients are severe visceral edema, refractory shock, and multiple organ dysfunction syndrome (MODS), all of which are triggered by endothelial barrier disruptions and vascular permeability changes. However, it is still unclear whether the HTNV infection causes glycocalyx destruction of vascular endothelial cells in patients with HFRS.

In this study, we observed the dynamic changes of the major degradation and shedding components of the glycocalyx, including HS, HA, and CS, during the clinical course in HFRS patients. Previous studies have shown that several pathological conditions, such as sepsis, diabetes, chronic and acute kidney injury, direct/indirect lung injury, ischemia/reperfusion, atherosclerosis, and inflammation are all associated with increased circulating HA, HS, and CS (29–34). HFRS has the basic clinical characteristics of sepsis, but it also has unique pathophysiological features such as the hypotensive phase of HFRS, which usually occurs between days 3 and 7 of the clinical course, and severe HFRS patients can manifest a more severe leukemoid reaction, increased vascular endothelial permeability, and coagulation dysfunction and usually have complications such as renal, cardiac, pulmonary, and hepatic injuries (3, 5, 6, 35, 36). In this study, the levels of plasma HS, HA, and CS of HFRS patients during the acute stage were all significantly higher than those of the healthy controls and of patients in the convalescent stage, which indicated that increased HS, HA, and CS could reflect the degradation and shedding of the glycocalyx under a systemic inflammatory response in HFRS patients and was similar to the circumstances of severe sepsis and septic shock (15, 30–32, 34). Furthermore, the levels of plasma HS and CS increased during the acute stage, and disease aggravation was higher in patients with more severe diseases and non-survivors, which also showed significant correlations with the length of hospital stay and conventional laboratory parameters. The abovementioned results demonstrated that the levels of HS and CS during the acute stage were significantly correlated with the severity of HFRS, which might also be beneficial to prognosis prediction.

Our previous studies have shown that the clinical course of patients with severe HFRS is usually associated with high levels of WBC, long APTT, and low levels of PLT and Fib (5, 6, 8). Furthermore, long-temporal tissue hypoperfusion and hypoxia on the liver and heart also induced a massive release of AST from the cytoplasm to the circulation and were also accompanied by synthesis dysfunction of ALB and extravascular leakage (5, 6, 8). In this study, we also observed that HS and CS were closely correlated with WBC, PLT, AST, ALB, blood urea nitrogen (BUN), APTT, Fib, and the length of hospital stay, which further proved that the destruction of the glycocalyx was accompanied by a more reactive inflammatory response, increased vascular endothelial permeability, and coagulation dysfunction, which might reflect the extent of injury to multi-organ function and disease severity to a degree. In addition, HS and CS were significantly associated with the death of HFRS in the Kaplan–Meier survival analysis, both of which also demonstrated obvious predictive efficiency for the prognosis (death) of HFRS by the ROC analysis. Interestingly, the expression of heparanase in the urine, plasma, and podocytes of glomerular capillaries was also increased in PUUV-infected patients and was strongly associated with the markers of severity (such as AKI and proteinuria) in HFRS patients (37). Both of these findings also indicated that there may be destruction and shedding of the glycocalyx in HFRS patients. Therefore, high expression of exfoliated glycocalyx fragments in plasma could reflect the severity of the vascular endothelial injury and microvascular leakage. Furthermore, we also found that HS and CS levels may be more diagnostically suitable for clinicians to evaluate the prognosis, which holds merit for future clinical application.

Despite the statistically significant results of our study, some limitations need to be acknowledged. First, this study was conducted at a single department for infectious diseases, and the relatively small sample size of the enrolled patients might result in poor statistical power. Only 13 critical-type deceased patients were enrolled, which likely influenced the result of the ROC curve analysis. Second, the major reason for the relatively higher fatality rate of patients with HFRS in this study is that, as a tertiary hospital, the patients treated in our center manifested more serious conditions and more severe clinical types on admission. Most of the patients with HFRS had been treated in primary hospitals for several days during the acute stage, and the patients with severe disease and poor curative effect were transferred to our hospital, which was also usually accompanied by uncontrolled shock and multi-organ dysfunction, thereby increasing the treatment difficulty. Consequently, the results of this study might be limited by these adverse factors. Third, we only defined the acute and convalescent stages in this study; therefore, the timing from disease onset to sampling collection was not unified or precise. Although there was no significant statistical difference in the timing of blood sampling during the acute stage, the levels of plasma HS, HA, and CS were influenced by this diversity. Furthermore, the prognosis and clinical classifications of the enrolled patients might be biased given the lack of a more standardized protocol for the management of HFRS. Therefore, it is essential to conduct a prospective, large-sample, multicenter cohort study to further confirm the predictive efficacy and clinical application value of HS, CS, and HA. Finally, we did not clarify the specific mechanism of glycocalyx destruction and the role of exfoliated glycocalyx components in the subsequent inflammatory response and the immunopathological injury during the acute stage of HFRS. Therefore, elucidating the mechanism of glycocalyx destruction and searching for the trigger or key enzymes involved in the regulation and degradation of the glycocalyx in HFRS may be the focus of future research. Exploring novel targets that can attenuate glycocalyx degradation or restore its integrity may be one of the important pharmacological interventions to improve vascular permeability in HFRS and other viral diseases with “microvascular leakage.”

In conclusion, the destruction and shedding of the glycocalyx may be closely associated with endothelial hyperpermeability and microvascular leakage in HFRS. Dynamic detection of the exfoliated glycocalyx fragments may be beneficial to evaluate the disease severity and prognosis in HFRS and to help clinicians implement an optimal therapeutic regimen in a timely, precise, and individualized manner.

## Data availability statement

The raw data supporting the conclusions of this article will be made available by the authors, without undue reservation.

## Ethics statement

The studies involving human participants were reviewed and approved by the Institutional Review Board of the Second Affiliated Hospital of Air Force Medical University. The patients/participants provided their written informed consent to participate in this study.

## Author contributions

HD and PW designed the study. HD, HH, and JL wrote the manuscript and collected the clinical data. HD and HH analyzed the data. JL, XW, and HJ performed most of the experiments and recruited the participants. JL, YZ, and PW supervised the study, revised the manuscript, and obtained funding. All authors contributed to the article and approved the submitted version.

## Funding

The study was supported by the Key Clinical Research Project of Technology Innovation and Development Foundation of the Second Affiliated Hospital of Air Force Medical University (2019LCYJ002), the General Clinical Research Project of Air Force Medical University (2021LC2222), the Key Support Project in Hygiene and Health of Shaanxi Province (2022A011), and the Routine Clinical Research Program of the Second Affiliated Hospital of the Air Force Medical University (No. 2021LCYJ025). The funders had no role in the design and conduct of the study or the preparation of the manuscript.

## Conflict of interest

The authors declare that the research was conducted in the absence of any commercial or financial relationships that could be construed as a potential conflict of interest.

The reviewer ZS declared a shared parent affiliation with the Authors to the Handling editor at the time of review.

## Publisher’s note

All claims expressed in this article are solely those of the authors and do not necessarily represent those of their affiliated organizations, or those of the publisher, the editors and the reviewers. Any product that may be evaluated in this article, or claim that may be made by its manufacturer, is not guaranteed or endorsed by the publisher.
